# How Do Nursing Students Use Digital Tools during Lectures?

**DOI:** 10.1371/journal.pone.0165714

**Published:** 2016-11-03

**Authors:** Isabelle Sebri, Jean-Claude Bartier, Thierry Pelaccia

**Affiliations:** 1 Saint-Vincent Nursing School, Strasbourg, France; 2 Emergency Departments Network in Alsace (RESURAL), Strasbourg, France; 3 Centre for Training and Research in Health Sciences Education (CFRPS), Faculty of Medicine, University of Strasbourg, Strasbourg, France; 4 Prehospital Emergency Care Service (SAMU 67), Strasbourg University Hospital, Strasbourg, France; Waseda University, JAPAN

## Abstract

**Objectives:**

Teachers often wonder what students are doing during lectures, behind their computers, mobile phones and other digital tools. This study aimed to document the type of tools used during lectures by nursing students and what they do with them.

**Methods:**

We carried out a descriptive, prospective, multicentre study including 1446 nursing students in Alsace (France). The students filled in an anonymous questionnaire at the end of a lesson they had just attended.

**Results:**

99% of the students had taken at least one digital tool to the lesson. 90% had a mobile phone with them. It was mainly used for entertainment (particularly for sending and/or receiving text messages and consulting emails). 52% had a laptop with them. It was essentially used for academic tasks (taking notes, working on other teaching units or revising for exams).

**Conclusion:**

Most nursing students take a phone or laptop to lectures with them with the intention of using them for entertainment and learning respectively. These results could guide training establishments in drafting their institutional policy concerning the use of digital tools in class.

## Introduction

The concept of "Digital Natives" has gained in popularity in recent years. Developed particularly by Prensky, it groups students born between 1978 and 1994 (who belong to "generation Y") and those born after 1994 (who belong to "generation Z") [1]. The young people in this generation form the largest number of students in health sciences training. Often opposed to their parents called "Digital Immigrants", they have used digital tools since childhood. Considered to be hyperconnected and multitaskers, they use digital tools massively, particularly smartphones, tablets and laptops, including in a learning environment [2–5]. This massive use is thought to have consequences on their behavior [5]. A recent study revealed that 25% of students systematically use a computer during lessons [6]. This has particularly replaced paper and pen for taking notes in class [4].

But which teacher has not wondered what really goes on behind the screen and whether the students are really using it in relation to the course or not? This question is all the more important that recent research has shown that using digital tools in class can be prejudicial, particularly when it has nothing to do with the lesson [5]. This effect has also been observed in the immediate neighbours of the students concerned, who register a significantly altered performance level [5].

To our knowledge, no work has been done to document the actual use made of digital tools by health sciences students during their classes. This study had a dual objective:

- To identify the type of digital tools that nursing students use during lessons.
- To accurately document the use they make of these tools during lessons.

## Materials and Methods

### Study population

The target population was composed of nursing students in Alsace, in Eastern France. In France, studies leading to a State nursing diploma take place in 6 training semesters, alternating practical and theoretical training. Half of the theoretical training is in the form of lectures, with attendance mandatory or not.

### Study design and questionnaire design

We emailed requests to the directors of the 10 training institutes in Alsace. Eight establishments agreed to participate. All the students at these institutes were included in the study, for a total of 1674 students. The data were collected between February and April 2015, via an anonymous self-administered questionnaire split into five parts:

- A first part giving a brief description of the study objectives and offering the students the opportunity not to participate.
- A second part questioned the students on how they took notes during the lecture
- A third part was on the digital tools students had with them during the lecture
- A fourth part was on how these tools were used during the lecture and how often they were used
- A fifth part collected sociodemographic data such as age, gender, the study semester number and the type of lessons attended.

The questionnaire was validated by a group of experts for its content validity. It was subsequently validated with a group of nursing students to be sure that the questions were well understandable.

A single lesson was targeted at random, for each student year. At the end of the lesson the questionnaires were distributed to the students and taken in immediately. The students were given the choice not to fill in the questionnaire if they didn't want to take part in the survey. Our study has been approved by the ethics committee of the faculty of medicine of Strasbourg.

## Results

1446 students were present at the lectures used to collect the data. They all filled in the questionnaire. 85.3% were women and 14,7% men. The average age was 24.1 (standard deviation: 6.43 years).

### Lecture and classes characteristics

The lectures used as support for the study lasted between 1 and 3 hours. Of the 16 lectures, only 2 were given by instructors who are permanent members of the nurse-training school staff. The other classes were taken by outside instructors.

The size of each student group varied from 42 to 162 students (average: 90.38; standard deviation: 40.26). Seven classes out of the 16 were mandatory and all—except one—included a digital support such as a slide show.

### Taking notes during the lesson

49.8% of the students took notes on paper and 28.5% on their laptop or tablet. 5.7% took notes on both of these. 16% of the students did not take notes.

### Possession and use of digital tools during the lesson

#### Mobile phone

90.5% of the students had a phone, generally a smartphone (77%). Of these:

- 62% had used it for performing tasks not linked to the lesson. These included sending text messages (60,8%), reading and answering email (27.57%), managing their diary (23.83%), chatting on social media (20.2%), searching the Internet for information not linked to the lesson (20%), and/or playing games (16.6%). More rarely, they were taking photos, using Snapchat, looking up the time and downloading applications.
- 18.7% had used it for on-line activities linked to learning. It involved searching the Internet for information linked to the lesson (14.2%) or taking notes (4,5%).
- 20.8% said that they did not use their phone during the lesson.

#### Laptop and tablet

52.5% of the students had either a laptop (mainly) or a tablet. Of these:

- 80.3% had used it for learning purposes. These were essentially taking notes linked to the lesson (53%), working for other teaching units (12.8%), searching for information on the Internet on the subject of the lesson (8%) and/or revising for exams (7.4%).
- 33.9% had used it to perform tasks not linked to the lesson. These were mainly reading and answering emails (9.6%), searching the Internet for information not linked to the lesson (7.1%), playing games (5.2%), chatting on social media (4.9%) and/or sending text or multimedia messages (3.8%).
- 11.9% stated that they had not used their laptop or tablet during the lesson.

## Discussion

As was shown by Prensky, the Digital Natives included in our study used digital tools massively during the main lesson, for various reasons [1].

### The laptop and tablet for learning activities

The laptop and tablet were essentially used by the students for learning, as an alternative to taking handwritten notes, which, however, were used by the majority in our study population. On the controversial subject of note taking, it should be noted that a recent study has shown that taking handwritten notes is more efficient than taking notes on a computer [4]. Writing notes with a pencil on paper is known to stimulate memorising of information and structuring of knowledge [4–7]. Students who take notes on a computer collect more information than those who use handwriting, but they are more content to take down what the teacher is saying, word for word, which is not an efficient learning activity [4]. Therefore, the pertinence of using a laptop or table to take notes during a lesson is questionable from the point of view of the quality of learning achieved.

### The mobile phone for entertainment

The many students who brought their mobile phone into class used it mainly for performing tasks not connected with the lesson or their training. Because of this, it seems legitimate, if there is an institutional policy concerning the use of digital tools, to prohibit the use of mobile phones during lessons. However, it would also be possible to use smartphones as teaching and learning tools.

For most of them, technologies used for teaching and learning purposes were not originally intended for such use [8]. They have been diverted from their original use to perform teaching activities more efficiently or faster [8]. With this in mind, smartphones could be used at the teacher's request for purposes of learning, e.g. as electronic ballot boxes to explore the students' prior knowledge, carry out formative or summative assessments during or at the end of the lesson, or to give students who do not want to express themselves orally, the chance to ask questions. The advantages of such activities are to provide immediate feedback about the learning process, both to learners and teachers [9]. In regard to the contemporary principles of adult learning, these activities are also considered as efficient means to enhance students’ active participation to knowledge building [10], and to facilitate the integration of new concepts with prior knowledge [11]. They also contribute to promote interactions between peers—provided that students are given the opportunity to discuss their answers—and with their teachers [10]. All these observations explain why these activities are likely to lead to positive outcomes in terms of learning, and to improve the performance levels reached by the students [10].

Using smartphones as boxes has the advantage of limiting the investment required in buying ballot boxes specifically designed for the purpose. The economic cost of information and communication technologies in education is known as one of the main barriers for the introduction of these technologies in everyday teaching practice [10]. Using smartphones for this purpose could also lead students to perceive the related learning activities as even more entertaining than when using ballot boxes, which may in turn enhance their cognitive engagement in these activities [12].

### Strengths and weaknesses of the study

Our study had a certain number of limits. Although the population included was quantitatively very large, which increased the external validity of our study, the fact that the students were questioned in their training establishment may have led them to give the answers expected by the institution, which do not necessarily reflect their actual behaviour. The anonymity of the questionnaires was intended to limit this risk. Moreover, the use of a self-administered questionnaire can be criticized with respect to the type of behaviours studied. Alternatives could have been chosen, such as observation and interviews. We considered that they were subject to more limitations than collecting data via questionnaires.

## Conclusions

To our knowledge, this is the first study to explore the way health sciences students—in our case, nursing students—use their digital tools during lectures. The methods we used only give a partial view of these behaviours and do not lead to any particular understanding of the sense attributed by the subjects concerned. In this context, we plan to continue this study with semi-structured interviews, with a grid built on the basis of the results presented in this article.

## Supporting Information

S1 File Questionnaire EN. Questionnaire used for the study (in English).
(DOCX)

S2 File Questionnaire FR. Questionnaire used for the study (in French).
(DOCX)

S3 File Data (in English). Data collected in this study (in English).
(XLSX)

S4 File Data (in French). Data collected in this study (in French).
(XLSX)
