# Supplementary material for: How Do Nursing Students Use Digital Tools during Lectures?
Source: PLoS One. 2016 Nov 3;11(11):e0165714. doi: 10.1371/journal.pone.0165714 (PMC5094670; doi:10.1371/journal.pone.0165714)
Supplement: S1 File — Questionnaire used for the study (in English). (DOCX) [file pone.0165714.s001.docx]

**How you use digital tools in class**

Hello,

We are inviting you to take part in a study being carried out by the Strasbourg Faculty of Medicine as part of its Masters in Education programme. The purpose of this study is to understand the ways in which you use digital tools (computers, mobile telephones, tablets, etc.) during classes. The questionnaires are entirely **anonymous** and your teachers will not be able to see your answers. We would be grateful that you answer the questions as honestly as possible in order to produce a valid study that will allow us to improve teaching conditions for you. If you do not want to take part in the study, just return the questionnaire blank.

Thank you very much for your contribution.

Isabelle Sebri, Masters Student in Health Sciences Education

Thierry Pelaccia, Teacher-Researcher at Strasbourg Faculty of Medicine

**Taking notes during classes**

**Q1- For this class, you took notes**

| Only on paper | Only on a computer and/or tablet | Both on paper and a digital device | I did not take notes |
| --- | --- | --- | --- |
| 🌕 | 🌕 | 🌕 | 🌕 |

**Q2- During this class, you supplemented your note-taking with** *(more than one option possible)***:**

| Q2-1 | A discussion with your neighbours | 🌕 |
| --- | --- | --- |
| Q2-2 | A document search on the internet | 🌕 |
| Q2-3 | A live discussion on a forum | 🌕 |
| Q2-4 | Reading a textbook or other publication | 🌕 |
| Q2-5 | Other (*specify*): | 🌕 |
| Q2-6 | I did not supplement my note-taking | 🌕 |

**How you used digital tools DURING THIS CLASS**

**Q3- Which digital tools did you have with you during this class?** *(more than one option possible)*

| Regular mobile telephone | 🌕 no | 🌕 yes, on the table | 🌕 yes, in my bag or my pocket |
| --- | --- | --- | --- |
| Smartphone | 🌕 no | 🌕 yes, on the table | 🌕 yes, in my bag or my pocket |
| Tablet | 🌕 no | 🌕 yes, on the table | 🌕 yes, in my bag or my pocket |
| Computer (all types: laptop, hybrid) | 🌕 no | 🌕 yes, on the table | 🌕 yes, in my bag or my pocket |

**Q4- During this class (excluding any breaks),** you used your telephone to *(more than one option possible)*:

| Search the internet for information I needed relating to the class | 🌕 |
| --- | --- |
| Search the internet for information not relating to the class | 🌕 |
| Make a telephone call | 🌕 |
| Send SMS/MMS | 🌕 |
| View and/or reply to emails | 🌕 |
| Stream TV/videos | 🌕 |
| Download applications | 🌕 |
| Post on social networks | 🌕 |
| Take photos | 🌕 |
| Play games | 🌕 |
| Take notes | 🌕 |
| View your diary or timetable | 🌕 |
| Other (*specify*): | 🌕 |
| I did not use my mobile telephone during this lesson | 🌕 |

| Q4-15 | **not applicable** (I did not have my mobile telephone with me)  **🡪 go straight to question Q7** | 🌕 |
| --- | --- | --- |

| Q4-16 | **I don't want to answer** | 🌕 |
| --- | --- | --- |

**Q5-** How often did you use your telephone **DURING** this class (excluding any breaks) **to take notes or search the internet for information relating to the class**?

| **Never** | **Once** | **Several times** | **Often** | **All the time** | **I don't know** |
| --- | --- | --- | --- | --- | --- |
| 🌕 | 🌕 | 🌕 | 🌕 | 🌕 | 🌕 |

| **I don't want to answer** | 🌕 |
| --- | --- |

**Q6-** How often did you use your telephone **DURING** this class (excluding any breaks)  **to do something** other than take notes or search the internet for information relating to the class?

| **Never** | **Once** | **Several times** | **Often** | **All the time** | **I don't know** |
| --- | --- | --- | --- | --- | --- |
| 🌕 | 🌕 | 🌕 | 🌕 | 🌕 | 🌕 |

| **I don't want to answer** | 🌕 |
| --- | --- |

**Q7- During this class (*excluding any breaks*)**, you used **your tablet and/or computer** to *(more than one option possible)*:

| Q7-1 | Search the internet for information I needed relating to the class | 🌕 |
| --- | --- | --- |
| Q7-2 | Search the internet for information not relating to the class | 🌕 |
| Q7-3 | Post on social networks | 🌕 |
| Q7-4 | View and/or reply to emails | 🌕 |
| Q7-5 | Stream TV/videos | 🌕 |
| Q7-6 | Download applications | 🌕 |
| Q7-7 | Send SMS/MMS | 🌕 |
| Q7-8 | Take photos | 🌕 |
| Q7-9 | Play games | 🌕 |
| Q7-10 | Do work for other modules | 🌕 |
| Q7-11 | Rewrite class notes | 🌕 |
| Q7-12 | Revise | 🌕 |
| Q7-13 | Other (*specify*): | 🌕 |
| Q7-14 | I didn't use my computer or tablet during this class | 🌕 |

| Q7-15 | **not applicable** (I didn't have my tablet or computer with me)  **🡪 go straight to question Q10** | 🌕 |
| --- | --- | --- |

| Q7-16 | **I don't want to answer** | 🌕 |
| --- | --- | --- |

**Q8-** How often did you use your tablet and/or computer **DURING** this class (excluding any breaks) **to take notes or search the internet for information relating to the class**?

| **Never** | **Once** | **Several times** | **Often** | **All the time** | **I don't know** |
| --- | --- | --- | --- | --- | --- |
| 🌕 | 🌕 | 🌕 | 🌕 | 🌕 | 🌕 |

| **I don't want to answer** | 🌕 |
| --- | --- |

**Q9-** How often did you use your tablet and/or computer **DURING** this class (excluding any breaks) **to do something** other than take notes or search the internet for information relating to the class?

| **Never** | **Once** | **Several times** | **Often** | **All the time** | **I don't know** |
| --- | --- | --- | --- | --- | --- |
| 🌕 | 🌕 | 🌕 | 🌕 | 🌕 | 🌕 |

| **I don't want to answer** | 🌕 |
| --- | --- |

| MALE | 🌕 |
| --- | --- |
| FEMALE | 🌕 |

**Q10- You are:**

**Q11- You are aged:**

| **Under 20** | **Between 20 and 36** | **Over 36** |
| --- | --- | --- |
| 🌕 | 🌕 | 🌕 |

**Thank you for your contribution.**
