# Supplementary material for: How Do Nursing Students Use Digital Tools during Lectures?
Source: PLoS One. 2016 Nov 3;11(11):e0165714. doi: 10.1371/journal.pone.0165714 (PMC5094670; doi:10.1371/journal.pone.0165714)
Supplement: S2 File — Questionnaire used for the study (in French). (DOCX) [file pone.0165714.s002.docx]

**Votre utilisation des outils numériques en cours**

Bonjour,

Nous vous proposons de participer à une étude réalisée par la faculté de médecine de Strasbourg dans le cadre d’un master de pédagogie. Cette étude vise à comprendre l’usage que vous faites des outils numériques (ordinateur, téléphone portable, tablette, etc.) pendant le cours. Les questionnaires sont entièrement **anonymes** et vos enseignants n’auront pas connaissance des réponses. Nous vous remercions par avance de bien vouloir répondre avec le plus de sincérité possible aux questions qui vous seront posées, afin que l’étude réalisée soit valide et que nous puissions améliorer les conditions de votre formation. Si vous ne souhaitez pas participer à l’étude, il suffit de rendre le questionnaire vierge.

Merci beaucoup de votre participation.

Isabelle SEBRI, étudiante en master de pédagogie en sciences de la santé

Thierry PELACCIA, enseignant chercheur à la faculté de médecine de Strasbourg

**La prise de notes pendant le cours**

**Q1- Pour ce cours, vous avez pris des notes**

| Uniquement sur support papier | Uniquement sur l’ordinateur et/ ou la tablette | A la fois sur support papier et support numérique | Je n’ai pas pris de notes |
| --- | --- | --- | --- |
| 🌕 | 🌕 | 🌕 | 🌕 |

**Q2- Pendant ce cours, vous avez complété la prise de notes par** *(plusieurs réponses possibles)***:**

| Q2-1 | Une discussion avec vos voisins | 🌕 |
| --- | --- | --- |
| Q2-2 | Une recherche de documents sur internet | 🌕 |
| Q2-3 | Un échange en direct sur un forum | 🌕 |
| Q2-4 | La lecture d’un manuel ou d’un ouvrage | 🌕 |
| Q2-5 | Autre (*précisez*) : | 🌕 |
| Q2-6 | Je n’ai pas complété la prise de notes | 🌕 |

**Votre utilisation des outils numériques PENDANT CE COURS**

**Q3- Quels sont les outils numériques que vous aviez avec vous pendant ce cours?** *(plusieurs réponses possibles)*

| Téléphone portable classique | 🌕 non | 🌕 oui, sur la table | 🌕 oui, dans mon sac ou dans ma poche |
| --- | --- | --- | --- |
| Smartphone | 🌕 non | 🌕 oui, sur la table | 🌕 oui, dans mon sac ou dans ma poche |
| Tablette | 🌕 non | 🌕 oui, sur la table | 🌕 oui, dans mon sac ou dans ma poche |
| Ordinateur (tous les types : portable, hybride) | 🌕 non | 🌕 oui, sur la table | 🌕 oui, dans mon sac ou dans ma poche |

**Q4- Pendant ce cours (en dehors des temps de pause éventuels),** vous avez utilisé votre téléphone pour *(plusieurs réponses possibles)*:

| Chercher sur internet des informations qui me manquaient au sujet du cours | 🌕 |
| --- | --- |
| Chercher sur internet des informations sans rapport avec le cours | 🌕 |
| Téléphoner | 🌕 |
| Envoyer des SMS/MMS | 🌕 |
| Consulter et/ou répondre à des e-mails | 🌕 |
| Regarder la TV/ des vidéos en streaming | 🌕 |
| Télécharger des applications | 🌕 |
| Echanger sur les réseaux sociaux | 🌕 |
| Faire des photos | 🌕 |
| Jouer à des jeux | 🌕 |
| Prendre des notes | 🌕 |
| Consulter votre agenda, votre planning | 🌕 |
| Autre  (*précisez*) : | 🌕 |
| Je n’ai pas utilisé mon téléphone portable pendant ce cours | 🌕 |

| Q4-15 | **non concerné** (je n’avais pas avec moi de téléphone portable)  **🡪 passer directement à la question Q7** | 🌕 |
| --- | --- | --- |

| Q4-16 | **Je ne souhaite pas répondre** | 🌕 |
| --- | --- | --- |

**Q5-** A quelle fréquence, avez-vous utilisé votre téléphone **PENDANT** ce cours (en dehors des temps de pause éventuels) **pour prendre des notes ou chercher sur internet des informations au sujet du cours** ?

| **Jamais** | **Une seule fois** | **Quelques fois** | **Souvent** | **Tout le temps** | **Je ne sais pas** |
| --- | --- | --- | --- | --- | --- |
| 🌕 | 🌕 | 🌕 | 🌕 | 🌕 | 🌕 |

| **Je ne souhaite pas répondre** | 🌕 |
| --- | --- |

**Q6-** A quelle fréquence, avez-vous utilisé votre téléphone **PENDANT** ce cours (en dehors des temps de pause éventuels) **pour faire autre chose** que prendre des notes ou chercher sur internet des informations au sujet du cours?

| **Jamais** | **Une seule fois** | **Quelques fois** | **Souvent** | **Tout le temps** | **Je ne sais pas** |
| --- | --- | --- | --- | --- | --- |
| 🌕 | 🌕 | 🌕 | 🌕 | 🌕 | 🌕 |

| **Je ne souhaite pas répondre** | 🌕 |
| --- | --- |

**Q7- Pendant ce cours (*en dehors des temps de pause éventuels*)**, vous avez utilisé **votre tablette et/ ou votre ordinateur** pour *(plusieurs réponses possibles)*:

| Q7-1 | Chercher sur internet des informations qui me manquaient au sujet du cours | 🌕 |
| --- | --- | --- |
| Q7-2 | Chercher sur internet des informations sans rapport avec le cours | 🌕 |
| Q7-3 | Echanger sur les réseaux sociaux | 🌕 |
| Q7-4 | Consulter et/ou répondre à des e-mails | 🌕 |
| Q7-5 | Regarder la TV/ des vidéos en streaming | 🌕 |
| Q7-6 | Télécharger des applications | 🌕 |
| Q7-7 | Envoyer des SMS/MMS | 🌕 |
| Q7-8 | Faire des photos | 🌕 |
| Q7-9 | Jouer à des jeux | 🌕 |
| Q7-10 | Travailler pour d’autres U.E. | 🌕 |
| Q7-11 | Retranscrire des notes de cours | 🌕 |
| Q7-12 | Réviser | 🌕 |
| Q7-13 | Autre  (*précisez*) : | 🌕 |
| Q7-14 | Je n’ai pas utilisé mon ordinateur ou ma tablette pendant ce cours | 🌕 |

| Q7-15 | **non concerné** (je n’avais pas avec moi de tablette ou d’ordinateur)  **🡪 passer directement à la question Q10** | 🌕 |
| --- | --- | --- |

| Q7-16 | **Je ne souhaite pas répondre** | 🌕 |
| --- | --- | --- |

**Q8-** A quelle fréquence, avez-vous utilisé votre tablette, et/ ou votre ordinateur **PENDANT** ce cours (en dehors des temps de pause éventuels) **pour prendre des notes ou chercher sur internet des informations au sujet du cours** ?

| **Jamais** | **Une seule fois** | **Quelques fois** | **Souvent** | **Tout le temps** | **Je ne sais pas** |
| --- | --- | --- | --- | --- | --- |
| 🌕 | 🌕 | 🌕 | 🌕 | 🌕 | 🌕 |

| **Je ne souhaite pas répondre** | 🌕 |
| --- | --- |

**Q9-** A quelle fréquence, avez-vous utilisé votre tablette, et/ ou votre ordinateur **PENDANT** ce cours (en dehors des temps de pause éventuels) **pour faire autre chose** que prendre des notes ou chercher sur internet des informations au sujet du cours ?

| **Jamais** | **Une seule fois** | **Quelques fois** | **Souvent** | **Tout le temps** | **Je ne sais pas** |
| --- | --- | --- | --- | --- | --- |
| 🌕 | 🌕 | 🌕 | 🌕 | 🌕 | 🌕 |

| **Je ne souhaite pas répondre** | 🌕 |
| --- | --- |

| HOMME | 🌕 |
| --- | --- |
| FEMME | 🌕 |

**Q10- Vous êtes :**

**Q11- Vous avez :**

| **Moins de 20 ans** | **Entre 20 et 36 ans** | **Plus de 36 ans** |
| --- | --- | --- |
| 🌕 | 🌕 | 🌕 |

**Merci pour votre participation**
